# Nitrogen-Rich Polyacrylonitrile-Based Graphitic Carbons for Hydrogen Peroxide Sensing

**DOI:** 10.3390/s17102407

**Published:** 2017-10-21

**Authors:** Brandon Pollack, Sunshine Holmberg, Derosh George, Ich Tran, Marc Madou, Maziar Ghazinejad

**Affiliations:** 1 Department of Mechanical and Aerospace Engineering, University of California, Irvine, CA 92697, USA; pollackb@uci.edu (B.P.); sholmber@uci.edu (S.H.); deroshg@uci.edu (D.G.); mmadou@uci.edu (M.M.); 2 Irvine Materials Research Institute (IMRI), University of California, Irvine, CA 92697, USA; ictran@uci.edu; 3 Department of Mechanical Engineering, California State University, Fresno, CA 93740, USA

**Keywords:** hydrogen peroxide sensing, polyacrylonitrile, graphitization, electrospinning, graphitic nitrogen, pyridinic nitrogen

## Abstract

Catalytic substrate, which is devoid of expensive noble metals and enzymes for hydrogen peroxide (H_2_O_2_), reduction reactions can be obtained via nitrogen doping of graphite. Here, we report a facile fabrication method for obtaining such nitrogen doped graphitized carbon using polyacrylonitrile (PAN) mats and its use in H_2_O_2_ sensing. A high degree of graphitization was obtained with a mechanical treatment of the PAN fibers embedded with carbon nanotubes (CNT) prior to the pyrolysis step. The electrochemical testing showed a limit of detection (LOD) 0.609 µM and sensitivity of 2.54 µA cm^−2^ mM^−1^. The promising sensing performance of the developed carbon electrodes can be attributed to the presence of high content of pyridinic and graphitic nitrogens in the pyrolytic carbons, as confirmed by X-ray photoelectron spectroscopy. The reported results suggest that, despite their simple fabrication, the hydrogen peroxide sensors developed from pyrolytic carbon nanofibers are comparable with their sophisticated nitrogen-doped graphene counterparts.

## 1. Introduction

Hydrogen peroxide (H_2_O_2_) is a common byproduct of many biochemical reactions and is therefore a common analyte used to indirectly detect cellular signaling, aging mechanism, and various oxidases, such as glucose oxidase, NADPH oxidase, urate oxidase, and lactate oxidase [1,2,3,4,5,6]. Though its major application lies in biosensing, its detection is of high importance in various other fields including textile industry, the food industry, and paper manufacturing as well [7]. Consequently, the detection of H_2_O_2_ is a major field of research for many sensing related applications.

Various techniques have been employed to achieve sensing of H_2_O_2_ such as fluorescence, colorimetric, and electrochemical methods [1,2,3]. Among these, electrochemical detection, in general, has the reputation of having high sensitivity, selectivity, and affordability. However, early experiments of H_2_O_2_ sensing used noble metals, such as platinum and gold as catalytic surface for attaining high sensitivity, making the electrochemical detection of H_2_O_2_ an expensive option [8,9,10]. This has eventually led to the use of various carbon allotropes, such as pure graphene, carbon nanotubes, and graphite for this purpose, which not only circumvents the use of expensive noble metals but also allows for a non-enzymatic method of sensing [11,12,13,14,15]. Despite the advantages, the performances of these carbon allotropes are not on par with the existing sensors with noble elements. The main challenges in the area of carbon based H_2_O_2_ sensors include slow kinetics and interferences from species, such as ascorbic acid and uric acid, which prompts studies geared toward enhancing carbon catalytic performance [16].

In recent years, studies on the incorporation of heteroatoms into the carbon lattice have gained attention for its promise to enhance the catalysis of the modified carbon. The insertion of heteroatoms into the carbon matrices can tailor the electro-catalytic activity of the material to either enhance or diminish its electrons density. P-type graphene showed enhanced capability to oxidize, whereas the presence of n-type showed improved reduction capacity in various carbon allotropes [4,5,6]. The reducing nature of n-type carbon material, such as NRGC, is found to be highly advantageous in various applications, such as fuel cell and biosensors [17,18,19,20]. In particular, characterization of electrochemistry of nitrogen-rich graphitic carbons (NRGC) have revealed their potential as catalysts for various analytes such as hydrogen peroxide, ascorbic acid, dopamine, and uric acid [21]. Furthermore, NRGC’s enhanced electrocatalysis has been demonstrated to allow for the simultaneous detection of these analytes within the same solution [22]. Specifically, NRGC’s capacity for H_2_O_2_ reduction reaction is used as one of the standard measure of its catalytic performance [21].

Various techniques have been reported for the synthesis of nitrogen rich graphite, including doping methods such as chemical vapor deposition (CVD), ball milling, plasma treatment, arc discharge method, and thermal treatment [12,23,24]. Among these methods, CVD delivers controllable doping, whereas ball milling is known for its simplicity and scalability. However, CVD incurs excessive cost and low yield, while ball milling doping is difficult to control and is effective only at edges [12]. Nitrogen plasma treatment introduces as high as 8 at.% of nitrogen, but often leads to low electrocatalytic activity due to the damage that it causes on the carbon lattice [25]. Both arc discharge and thermal treatment methods only results in a very low nitrogen content (~1 at.%) [12]. These doping methods inherently involve multiple processing steps after synthesis of carbon platform. They often include introducing nitrogen into previously synthesized graphitic carbons by damaging the graphitic lattice for introducing holes and defects to allow for nitrogen assimilation. The nature of such a process is cumbersome and costly to control. Alternatively, there has been a growing interest in single step fabrication of nitrogen rich carbon using electrospun fibers [26]. The main challenge with this approach is that the microstructure of the resulting electrospun carbon fibers are reported to be mainly amorphous and glassy-like carbons, causing low conductivity and a reduced sensitivity.

In this study, we report on the H_2_O_2_ sensing capability of NRGC synthesized from pyrolysis of polyacrylonitrile (PAN). Polymer nanofibers are synthesized via electrospinning PAN solution, infused with multi-walled carbon nanotubes (MWCNTs). The as-spun polymer nanofibers are then mechanically compressed prior to and during the subsequent thermal stabilization step. The mechanical stresses introduced in the electrospinning process by the addition of MWCNTs further align the polymer chains within PAN, enhancing the extent of their graphitization during the pyrolysis [27]. This synthesis method allows for low-temperature graphitization of the PAN fibers, which consequently results in a significant amount of nitrogen groups to remain intact within the carbon microstructure. Raman spectroscopy, Transmission Electron Microscopy (TEM), and X-ray Photoelectron Spectroscopy (XPS) are conducted to gain insight into the NRGC’s microstructure. XPS analysis reveals a carbon microstructure with almost exclusively graphitic and pyridinic nitrogen groups. Electrochemical characterization is then performed to investigate the capacity of the formed NRGC for hydrogen peroxide reduction. The results signify a unique, cost-effective, and scalable approach to fabricate NRGC platforms for hydrogen peroxide detection.

## 2. Materials and Methods

### 2.1. Preparation of Electrospinning Solution

The Multi-walled carbon nanotubes (MWCNT) were dispersed in N, N-dimethylformamide (DMF 99.9%, Fisher Chemical, Fair Lawn, NJ, USA) under ultrasonication for 1 h at room temperature and kept stirring for 24 h to make 1% (*w*/*v*) MWCNT-DMF mixture. Pure polyacrylonitrile (PAN, M.W. = 150,000 g/mol, Sigma Aldrich, St. Louis, MO, USA) was mixed with MWCNT-DMF to produce 8% (*v*/*v*) PAN and 1% (*w*/*v*) MWCNT-DMF solution. The solution was then stirred at 40 °C for 24 h.

### 2.2. Synthesis of 8% PAN and 1% CNT Carbon Nanofibers Mats

The Carbon Nanofiber (CNF) mats were prepared via a far-field electrospinning technique followed by a stress-induced stabilization and carbonization process. The precursor solution was dispensed via a 1-mL syringe with a 21-gauge needle mounted on a syringe pump at a solution consumption rate at 0.7 mL/h. The operating electrical potential of 15 kV was applied across the needle and a target aluminum-foiled drum (8 cm × 8 cm). The electrospinning process was performed for approximately 1–2 h to produce a mat thickness of approximately 100 μm.

### 2.3. Stress-Induced Stabilization

Electrospun CNF mats were then manually rolled on Dayton’s DC Speed Control Roller for 10 times repeatedly to give the mat a uniformly-aligned surface prior to its thermal stabilization. Rolled mats were then mechanically stressed with 200 kPa pressure on a metal compression platform and were thermally stabilized at 280 °C for 6 h. Samples that underwent this stress-induced stabilization and have the CNT are referred to as Mechanically Treated CNF. The electrodes produced from pure PAN and processed without the mechanical treatment will be referred from here onward as CNF.

### 2.4. Pyrolysis

Stabilized nanofiber mats were then pyrolyzed in a tube furnace with a continuous flow of nitrogen at a rate of 4600 sccm (standard cubic centimeters per minute) during the process. The furnace was initially heated to 300 °C at a ramp rate of 4.5 °C/min and dwelled at that temperature for an hour. Followed by a ramp rate of 3 °C/min until it reached 1050 °C, the furnace was held at this temperature for 1 h before cooling to ambient temperature at a rate of 10 °C/min.

### 2.5. Electrochemical Characterization

**Figure 1:**
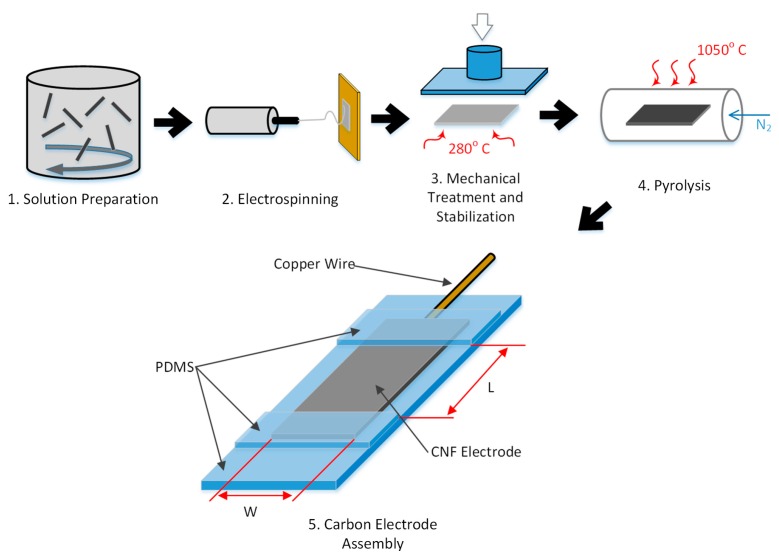
Synthesis process for mechanically treated polyacrylonitrile (PAN) Carbon Nanofiber (CNF) electrode for electrochemical characterization (note: Fabrication of untreated mats does not involve multi-walled carbon nanotubes (MWCNT) and mechanical compression in steps 1 and 3, respectively.)

A Princeton Applied Research VersaSTAT 4 Potentiostat running VersaStudio 2.48.5 was used for all of the electrochemical experiments. Figure 1 illustrates the CNF fabrication process and subsequent preparation steps for electrochemical characterization. The CNF mats are cut and then placed onto a cured sheet of Polydimethylsiloxane (PDMS, Sylgard 184 Silicone Elastomer, Dow Corning Corporation, Midland, MI, USA) for electrochemical characterization. Copper enamel coated wire is bonded onto the CNF electrode at the very edge with conductive carbon paint (Structure Probe, Inc., West Cherster, PA, USA). Two pieces of cured PDMS are then placed on the ends of each CNF electrodes and secured in place with uncured PDMS. Careful attention was paid to cover the wire bonded area completely with PDMS. The electrode assemblies are then placed on a hot plate so the PDMS would cure. This PDMS backbone allows the normally fragile CNF electrodes to be more durable, while the center section remains free. The geometric area of the CNF electrode is taken from the exposed length (L) and width (W) dimensions and calculated as a flat electrode.

For electrochemical characterization, a blank electrolyte solution of 1X Phosphate-buffered saline (PBS) was used. 1X PBS solutions are made by dissolving Disodium phosphate (Na_2_HPO_4_, Fisher Chemical), Monopotassium phosphate (KH_2_PO_4_, Sigma Aldrich, S.t. Louis, MO, USA), Sodium chloride (NaCl, Fisher Chemical) and Potassium chloride (KCl, Sigma Aldrich) in DI water. The target final concentrations of the 1X PBS was 0.14 M NaCl, 0.0027 M KCl, and 0.010 M PO_4_^3−^. The pH was adjusted to 7.4 with the addition of HCl or NaOH as necessary. The PBS solution was purged of oxygen via bubbling of nitrogen gas prior to the test. During the test, nitrogen gas was left flowing in the headspace above the liquid to help prevent any oxygen from dissolving back into the solution. All of the electrochemical tests were run while the solution was stirring. A Ag/AgCl electrode in saturated 3 M KCl solution was used as reference electrode and Glassy Carbon was used as counter electrode for all the electrochemical tests. Electrochemical Impedance Spectroscopy experiments (EIS) were performed in the 1X PBS solution to estimate active surface area from the capacitance of the electrodes [28]. Cyclic Voltammetry tests (CV) were performed in the 1X PBS with and without Hydrogen Peroxide (H_2_O_2_, 30% (*w*/*w*), Fisher Chemical). Chronoamperometry (CA) was conducted by incrementally increasing the concentrations of H_2_O_2_ to the final concentration of 2.5 mM. Interference tests were carried out by conducting chronoamperometry with Ascorbic Acid (AA, Sigma Aldrich), Glucose (Glu, EMD Millipore, Temecula, CA, USA) and Uric Acid (UA, Sigma Aldrich), as interfering agents.

## 3. Results

### 3.1. Raman Spectroscopy

**Figure 2:**
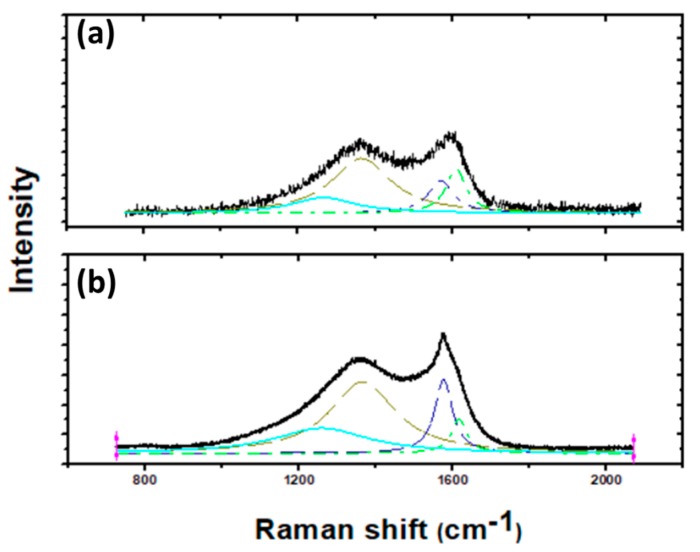
Comparison of peak intensities and peak width of Raman spectroscopy of (**a**) CNF and (**b**) Mechanically Treated CNF.

Raman spectroscopy is used as one of the major non-destructive techniques to inspect the degree of graphitization of a precursor after pyrolysis [29]. Here, we performed the characterization using a Renishaw InVia Raman Microscope with a 532 nm excitation laser. A four-peak Lorentzian fit was performed with peak locations occurring at 1220, 1355, 1583, and 1624 cm^−1^, which correspond to I, D, G, and D’, respectively [30,31]. Representative spectra of Mechanically Treated CNF and the CNF samples obtained from pure PAN without any mechanical treatment (CNF) are shown in Figure 2. We mainly examined the G and D peaks of the spectra, where the G peak ($I_{G}$) correspond to in-plane vibration ($E_{2g}$) and the D peak ($I_{D}$) is linked to the breathing mode of sp^2^ atoms due to structural defects. Subsequently, a ratio (R_D/G_) between $I_{D}$ and $I_{G}$ is used as a scale of graphitization [32].

The Raman spectra of CNF and mechanically treated CNF showed two major peaks, D and G, in the range of 1350–1370 and 1580–1590. The resulted spectra showed a noticeable improvement in graphitization for mechanically treated CNF sample in comparison with CNF as indicated by the reduced value of R_D/G_. The D peak to G peak ratio calculated from deconvoluted Raman spectra showed a reduction from an approximate value of 1.95 in the case of CNF to 1.0 for Mechanically Treated CNF. Formation of relatively larger nanocrystallines for mechanically treated CNF as compared to the CNF specimen is also evident from the sharp G peak in the mechanically treated sample [33]. The increased proportion of D (1355 cm^−1^) and D’ (1624 cm^−1^) peaks for untreated material indicate a high degree of disorders. Furthermore, the presence of peak at 1220 cm^−1^ shows the presence of foreign atoms [17]. A high percentage of pyridinic nitrogen could lead to the presence of more nanoholes inside the graphene layers [34]. On the other hand, the presence of graphitic nitrogen does not induce such defects (details of various types of nitrogen present in the carbon structure is discussed further ahead in the article). This, in conjunction with the XPS data showing high content of graphitic nitrogen, explains the reduced R_D/G_ value in case of mechanically treated CNF.

### 3.2. High-Resolution Transmission Electron Microscopy (HRTEM)

**Figure 3:**
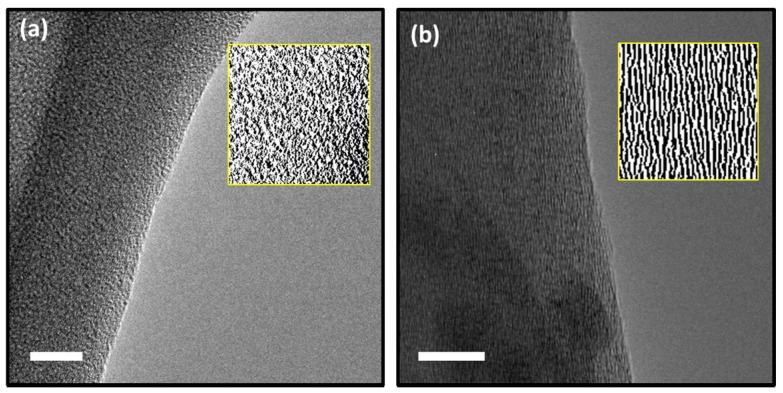
Transmission Electron Microscopy (TEM) images of carbon obtained from (**a**) Mechanically Treated CNF and (**b**) pure PAN with processed image in inset. The scale shown is 10 nm.

For further validation of the data obtained through Raman spectroscopy, High-Resolution Transmission Electron Microscopy (HRTEM) analyses were also conducted. The evaluation of fringes provides information about the degree of graphitization of the carbon mats. Here, the TEM images of carbon specimen, taken by FEI’s Titan S/TEM, are provided to comparatively study the microstructures of pure PAN-based CNF and mechanically treated CNF. The micrographs obtained from the pure PAN CNF using HRTEM demonstrates randomly oriented pattern, pointing towards the amorphous nature of the polymer (Figure 3a). On the other hand, the mechanically treated CNF showed crystalline nature as shown in Figure 3b. The TEM observations are consistent with the Raman spectra obtained from the mechanically treated CNF specimen.

### 3.3. X-ray Photoelectron Spectroscopy (XPS)

**Figure 4:**
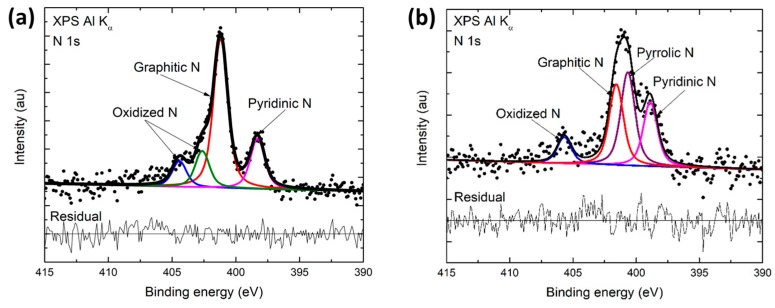
(**a**) Fitting N 1s X-ray photoelectron spectroscopy (XPS) peak of CNF, (**b**) Fitting N 1s XPS peak of mechanically treated CNF.

In order to obtain the elemental and compositional constituents of the final product of the pyrolysis, X-ray photoelectron spectroscopy was performed using a Kratos AXIS-SUPRA surface analysis instrument, equipped with a monochromatic Al Kα X-ray source. Data obtained from XPS showed that the mechanically treated CNF has 94.5 ± 0.1 at.% carbon, 4.4 ± 0.5 at.% nitrogen and 1 ± 0.3 at.% of oxygen content in it. The component of interest here is the presence of nitrogen, as it has been reported that nitrogen doped graphene sheets possess an increased H_2_O_2_ reduction capability contrasting pure graphene. Fitting high resolution N 1s XPS peaks shows the presence of pyridinic nitrogen (pyridinic-N), pyrrolic nitrogen (pyrrolic-N), and graphitic nitrogen (graphitic-N), with a relatively high content of pyridinic-N (19.2%) and graphitic-N (57%) in the mechanically treated material. In comparison, CNF without any mechanical treatment has significantly high content of pyrrolic-N (35.3%) (Figure 4a,b and Table 1).

It has been reported that the presence of pyridinic-N and graphitic-N enhances the catalytic behavior as it provides extra electron density to the graphitic basal plane [23]. The electron affinity possessed by nitrogen atoms causes a positive charge enhancement in the adjacent carbon atoms in the basal plane. This thought to be an adsorption enhancer in graphitic carbon [35]. This, consequently, aids the electrochemical reduction reaction. Moreover, a side-on adsorption of H_2_O_2_ (Yeager model) is made possible through this N-doping, making its O–O bond relatively weaker as compared to an edge-on adsorption, which in turn enables easier hydrogen peroxide reduction [36,37].

A high degree of graphitization with graphitic edges can also contribute towards an improved electrochemical performance. Hence, an increase in the proportion of graphitic-N in the carbon structure is advantageous. Graphitic-N has a higher thermal stability as compared to the other types of nitrogen present in the material. Therefore, higher relative concentrations of graphitic nitrogen are generally observed at higher pyrolysis temperatures [38]. It is important to note that the overall nitrogen content within the pyrolytic carbon decreases as the pyrolysis temperature increases. Consequently, we have two competing factors that influence the overall quantity of graphitic nitrogen in the pyrolytic carbon structure. Here, the application of mechanical treatment in the fabrication of carbon with relatively low-temperature pyrolysis has resulted in nitrogen content as high as 4.4%, of which 57% is graphitic nitrogen.

### 3.4. Electrochemistry

It has been shown that N-doped graphene sheets have higher electrocatalytic capacity for H_2_O_2_ reduction as compared to that of pure graphene [21]. This phenomenon is attributed to the presence of pyridinic-N and graphitic-N sites, which assist H_2_O_2_ reduction by weakening the O–O bond [35].

Additionally, it is expected that graphitic carbon structures with edge planes would demonstrate higher electrocatalytic activity as compared to amorphous carbons. In the previous characterizations, we demonstrated that our mechanically treated CNFs is inherently rich in graphitic edge planes, and possess high concentrations of pyridinic and graphitic nitrogen atoms. Thus, we anticipate that the synthesized carbons exhibit high propensity for H_2_O_2_ sensing owing to enhanced electrocatalytic reduction of hydrogen peroxide.

Electrochemical Impedance Spectroscopy (EIS) was performed on the carbon electrodes in a blank solution of 1X PBS (pH 7.4) to determine the electrochemically active surface areas. The results are shown as Nyquist plots and are presented with an equivalent electrical circuit in Supplementary Figure S1. The double layer capacitance (*C_dl_*) was determined from fitting the equivalent circuit model to the Nyquist plots [39]. The active surface area (*A_act_*) of the electrode was calculated using Equation (1) [28]:

(1) $$A_{act}=\frac{I_{c}}{C_{dl}*v}$$

Here, *C_dl_* is the double-layer specific capacitance (µF/cm^2^) and the scan rate is given as v (V/s). *I_c_* is the double layer charging current, acquired from cyclic voltammetry. The determined active surface areas were used to normalize the current responses for all of the electrochemical tests.

**Figure 5:**
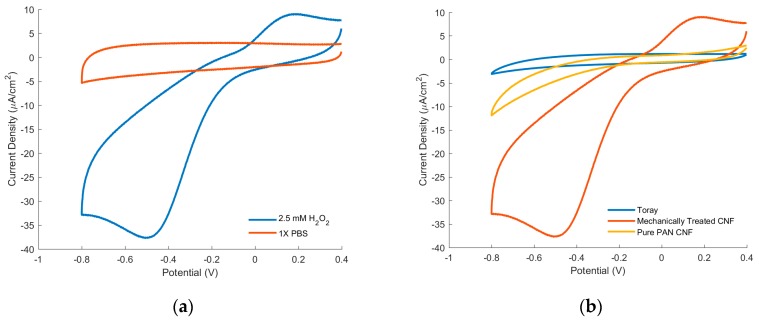
Cyclic voltammograms at 50 mV/s of (**a**) Mechanically treated CNF run in blank 1X PBS and in the presence of 1X PBS in the presence of 2.5 mM H_2_O_2_; (**b**) Toray, Mechanically Treated CNF, and pure PAN CNF electrodes in 1X PBS in the presence of 2.5 mM H_2_O_2._

Cyclic Voltammetry allows us to investigate the aptitude of mechanically treated CNFs for H_2_O_2_ reduction. In Figure 5, there are two sets of representative Cyclic Voltammograms (CV) which were run at 50 mV/s in 1X PBS (pH 7.4) with 2.5 mM of H_2_O_2_ (see Figure 5). All of the voltages are compared against Ag/AgCl. Figure 5a shows a comparison of the CVs of mechanically treated CNF in 1X PBS with and without the presence of 2.5 mM of H_2_O_2_. With the addition of the H_2_O_2_, there is a clear increase in the current response, exhibiting a 300 µA increase at −0.5 V vs. Ag/AgCl. Furthermore, the treated CNFs exhibit an onset potential of −0.1 V vs. Ag/AgCl, comparable to other nitrogen-doped graphene based sensors [25]. It is worth noting that there is a peak seen in plain 1X PBS of mechanically treated CNF, which could be a result of residual amounts of oxygen being reduced. This response will be further investigated in future works.

The CVs of mechanically treated CNF is compared with that of the electrodes made from Toray (a type of commercially available graphitic fiber), and untreated pure PAN based CNF (Figure 5b). Toray exhibited no noticeable increase in current response after the addition of hydrogen peroxide, indicating little to no electrocatalysis of hydrogen peroxide reduction. Between pure PAN CNFs and treated PAN CNFs electrodes, treated PAN CNF electrodes demonstrated much larger current response with the addition of hydrogen peroxide.

The reported electrochemical results echo previous studies on NRGC electrocatalysis of hydrogen peroxide. As seen from the XPS data, pure PAN CNF contains small amounts of pyridinic-N and graphitic-N, which contributes to its reduction of hydrogen peroxide. Toray, on the other hand contains no nitrogen groups due to the high temperatures used for its synthesis and therefore has no reduction of hydrogen peroxide. Between treated and untreated CNFs, the much larger current response to hydrogen peroxide could be largely attributed to the higher graphitization seen in the treated CNFs, which is known to contribute to enhance electron transfer efficiencies.

**Figure 6:**
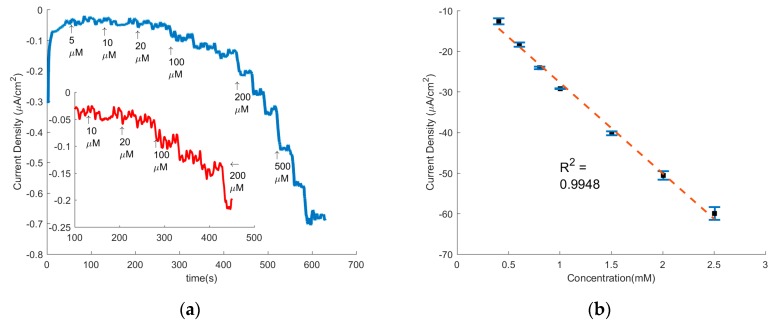
(**a**) Chronoamperometry of the Mechanically Treated CNF electrode running at −0.5 V. Each addition concentration is indicated on the graph and was repeated three times after the initial indication prior to the next concentration addition. The inset is a zoomed in portion of the main graph; (**b**) Concentration vs. Current Density data from the chronoamperometry fitted with linear regression trend line.

Chronoamperometry (CA) allows us to determine the sensitivity and limit of detection (LOD) of mechanically treated CNF as H_2_O_2_ sensor as seen by the representative CA graph in Figure 6a. The CA were performed at −0.5 V vs. Ag/AgCl, the typical potential at which the current density peaks for the treated PAN electrodes. During the CA, the current was allowed to stabilize before each subsequent additions of hydrogen peroxide and the concentration versus the current density was then extracted from the CA and plotted in Figure 6b.

From this Figure 6b, the sensitivity was calculated by the linear regression equation:

(2) I= −2.54C−0.604,

where *C* is the concentration in mM and *I* is the current density in µA cm^−2^. Here, the slope, and thus sensitivity, is 2.54 µA cm^−2^ mM^−1^ with a correlation coefficient (*R*^2^) of 0.9948. The LOD was then calculated by Equation (3), where a signal to noise ratio of 3 (S/N = 3) was used [40].

(3) $$LOD=\;\frac{3\sigma }{S}.$$

The standard deviation, σ, was taken from the stable signal of a blank solution and *S* is the linear regression equation’s slope. The LOD was calculated to be 0.609 µM. An average response time of 4.6 s was observed after each addition.

**Figure 7:**
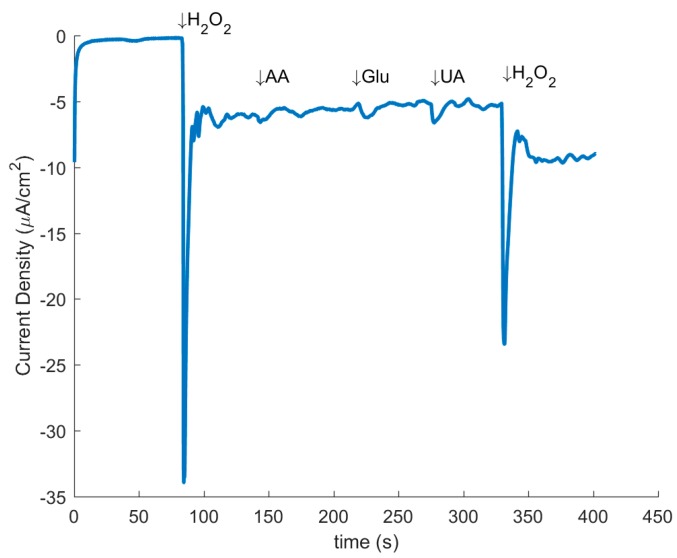
Amperometric response of the Mechanically Treated CNF at a potential of −0.5 V vs. Ag/AgCl with addition of interfering agents (1 mM H_2_O_2_, 0.15 mM AA, 1 mM Glu, 0.5 mM UA, and 1 mM H_2_O_2_).

In addition, CA was used to investigate the selectivity of the hydrogen peroxide reduction in the presence of other analytes, namely, Ascorbic Acid (AA), Uric Acid (UA), and Glucose (Glu). Figure 7 demonstrates the representative graph of CA with the resulting current responses after additions of 1 mM H_2_O_2_, 0.15 mM AA, 1 mM Glu, 0.5 mM UA, and a second 1 mM H_2_O_2_ at the indicated times. The results indicate that at the working potential of −0.5 V there was little to no response from any of the tested interfering agents, while there was still a noticeable response from the addition of H_2_O_2_. This signifies the high selectivity of the treated PAN electrodes to hydrogen peroxide reduction.

A number of works have reported application of different forms of carbon allotropes as a basis for H_2_O_2_ sensing with platforms such as nitrogen doped carbon nanotubes [41], graphene platinum nanocomposites [42], and nitrogen-doped graphene nanoribbons [43]. Such studies have resulted in sensitivities ranging between 0.967–154.78 µA mM^−1^, and LODs of 0.15 to 90 µM [43,44]. In comparison, the mechanically treated CNF sensors produce a sensitivity of 2.87 µA mM^−1^ when multiplied by active surface area. The acquired sensitivity suggests that, in spite of their facile synthesis route, the electrocatalytic performance of mechanically treated CNF sensors is comparable to that of the graphene-based sensors with more complex synthesis routes. Furthermore, the mechanically treated CNF demonstrated high selectivity towards H_2_O_2_ in the presence of the common interfering analytes: Glu, AA and UA. It is important to note that the H_2_O_2_ sensors were developed from as-pyrolyzed CNFs without additional processing. Refinement of the presented synthesis method will potentially yield additional enhancement in both the carbon graphitization degree and the quantity of nitrogen groups to further reduce the LOD, and augment the sensitivity of the electrodes.

## 4. Discussion

Nitrogen-rich graphitic carbons (NRGC) are emerging as an attractive platform for sensing devices. In particular, the presence of graphitic and pyridinic nitrogen in NRGC can enhance the catalytic behavior as it increases electron density of the graphitic basal planes. Accordingly, NRGCs have shown the capability of reducing substrates, such as H_2_O_2_, and have shown promise as potential alternatives to expensive metal-based catalysts such as gold. However, current methodologies for their synthesis such as nitrogen plasma bombardment, could damage the surface and morphology of the carbon structure. Here, we demonstrate an alternative synthesis method with which pyrolyzed graphitic substrates possessing N–C bonds can be produced in a single, facile pyrolysis step. Through the application of electrospinning technique, polymer fibers mats consisting of polyacrylonitrile (PAN) infused with carbon nanotubes (CNT) are produced and mechanically treated prior to their pyrolysis. The resulting pyrolytic carbon microstructure possesses a considerable increase in graphitization as demonstrated by its characterization using Raman spectroscopy, Transmission Electron Microscopy (TEM), and X-ray photoelectron spectroscopy (XPS). Furthermore, XPS analysis of the chemical constituents of the resulting carbon shows a high percentage of pyridinic-N and graphitic-N, which would enhance the reduction of hydrogen peroxide. Consistently, the mechanically treated CNFs showed a significant increase in the current response to hydrogen peroxide compared to untreated fiber mats as well as PAN based Toray. The treated carbon nanofiber mats were then characterized for their hydrogen peroxide sensing capability and demonstrated a sensitivity of 2.24 µA mM^−1^ and an LOD of 0.609 µM. The reported results suggest that, despite their facile fabrication, the hydrogen peroxide sensors developed from mechanically treated CNFs compare favorably to sophisticated nitrogen-doped graphene sensors. Thus, this report points to a cost-effective and scalable strategy for synthesizing nitrogen-rich graphitic carbons for hydrogen peroxide sensing.

## Figures and Tables

**Table 1:** Nitrogen species based on XPS analysis.

Component (BE)	at.% in CNF	at.% in Mechanically Treated CNF
Pyridinic-N (398.5 eV)	24.14	19.21
Pyrrolic-N (400.1 eV)	35.29	~0 *
Graphitic-N (401.3 eV)	30.58	56.97
Oxidized-N (405 eV & 402.6 eV **)	9.99 & ~0	13.97 & 9.85 **

* It appears that there is no pyrrolic-N formed in mechanically treated CNF; ** Another high BE peak is required to fit N 1s of mechanically treated CNF, which is also attributed to oxidized-N species. Very high resolution XPS might show evidence of oxidized pyridinic-N and oxidized graphitic-N that previous XPS works could not differentiate and ascribed generally to “oxidized N”. Other possibility could be “oxidized edge-N”

## Supplementary Materials

The following are available online at http://www.mdpi.com/1424-8220/17/10/2407/s1, Figure S1: Nyquist Plots of the Electrochemical Impedance Spectroscopy (EIS) for the Toray, Mechanically Treated CNF, and Pure PAN CNF electrodes. The equivalent electrical circuit is shown in the inset. In equivalent circuit, R_i_ represents the internal resistance, R_sf_ represents the surface resistance, C_sf_ represents the surface Capacitance, and R_ct_ represents the charge-transfer resistance. The double layer capacitance (C_dl_) is modeled as a Constant Phase Element (CPE) and diffusion affects are represented by the Warburg element (W), Table S1: Double layer capacitance, Geometric Surface Area, and Active Surface Area of electrodes, Figure S2: Wide XPS spectrum of the mechanically treated CNF, Figure S3: High resolution O 1s XPS spectra of mechanically treated CNF.
