# Supplementary material for: Nitrogen-Rich Polyacrylonitrile-Based Graphitic Carbons for Hydrogen Peroxide Sensing
Source: Sensors (Basel). 2017 Oct 21;17(10):2407. doi: 10.3390/s17102407 (PMC5676667; doi:10.3390/s17102407)
Supplement: Supplementary file 1 [file sensors-17-02407-s001.pdf]

sensors

Supplementary Information

Nitrogen-Rich Polyacrylonitrile-Based Graphitic Carbons for Hydrogen Peroxide Sensing

Brandon Pollack, Sunshine Holmberg, Derosh George, Ich Tran, Marc Madou and Maziar Ghazinejad

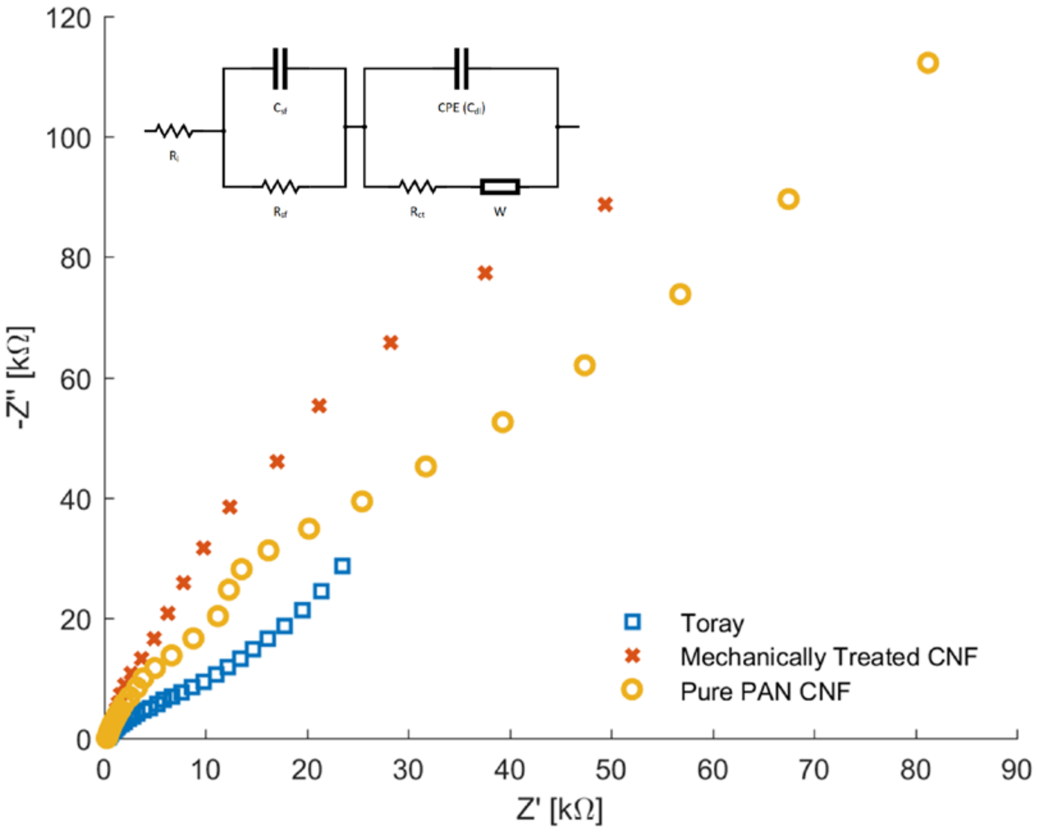

Figure S1. Nyquist Plots of the Electrochemical Impedance Spectroscopy (EIS) for the Toray, Mechanically Treated CNF, and Pure PAN CNF electrodes. The equivalent electrical circuit is shown in the inset. In equivalent circuit, R_i represents the internal resistance, R_{sf} represents the surface resistance, C_{sf} represents the surface Capacitance, and R_{ct} represents the charge-transfer resistance. The double layer capacitance (C_{dl}) is modeled as a Constant Phase Element (CPE) and diffusion affects are represented by the Warburg element (W).

Table S1. Double layer capacitance, Geometric Surface Area, and Active Surface Area of electrodes.

Electrode	C_{dl} (μF/cm2)	A_g (cm^2)	A_{act} (cm^2)
Pure PAN CNF	65.6	0.15	0.882
Mechanically Treated CNF	291.0	0.1	1.13

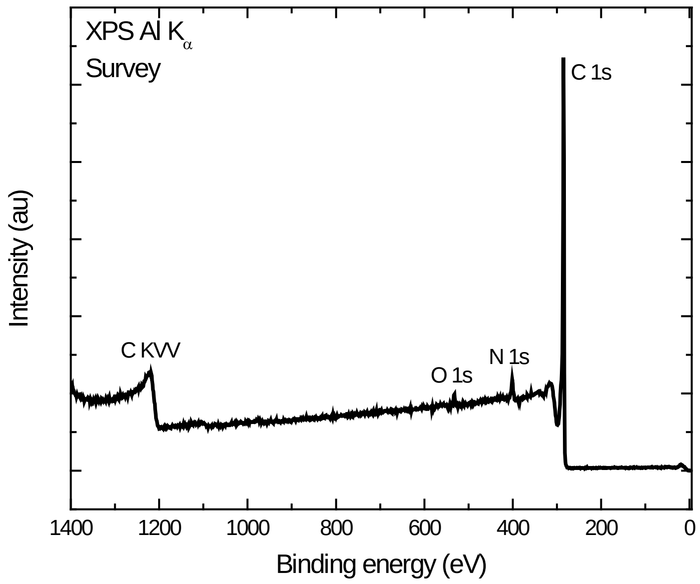

Figure S2. Wide XPS spectrum of the mechanically treated CNF.

It was observed that the percentage of oxygen (~1 at. %) in the sample is significantly small as compared to nitrogen (~4.5 at. %). Although the presence of oxygen may also influence the catalytic behavior of the material, the effect of nitrogen prevails [1]. The oxygen peak, deconvoluted for C–O, O–C=O and O-H, is shown in Figure S3.

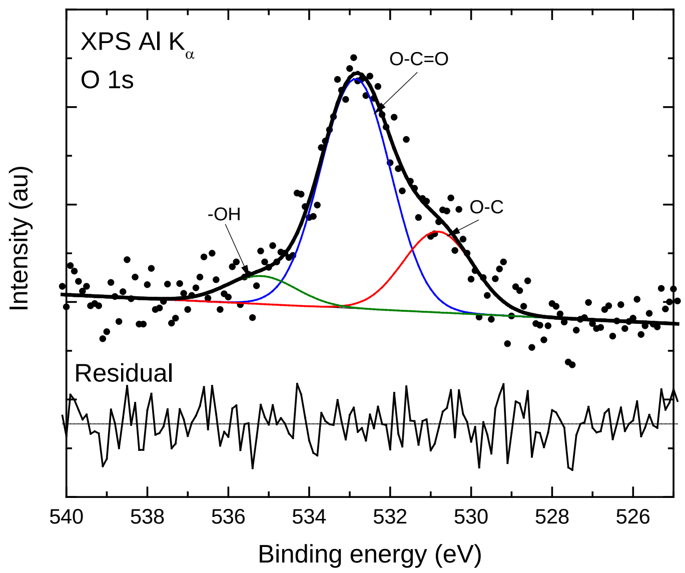

Figure S3. High resolution O 1s XPS spectra of mechanically treated CNF.

[1] Y. Shao, S. Zhang, M. H. Engelhard, G. Li, G. Shao, Y. Wang, J. Liu, I. A. Aksay, and Y. Lin, J. Mater. Chem. **20**, 7491 (2010).

© 2017 by the authors. Submitted for possible open access publication under the terms and conditions of the Creative Commons Attribution (CC BY) license (http://creativecommons.org/licenses/by/4.0/).
